# Caffeine mitigates tamoxifen-induced fatty liver in Wistar rats

**DOI:** 10.1590/acb396924

**Published:** 2024-09-30

**Authors:** Yasin Sezgin, Ejder Saylav Bora, Duygu Burcu Arda, Yiğit Uyanikgil, Oytun Erbaş

**Affiliations:** 1 Yüzüncü Yıl University – Faculty of Medicine – Clinic of Medical Oncology – Van – Turkey.; 2 İzmir Katip Çelebi University – Faculty of Medicine – Department of Emergency Medicine – Izmir – Turkey.; 3 Ege University – Faculty of Medicine – Department of Histology and Embryology – Izmir – Turkey.; 4 Taksim Research and Training Hospital – Department of Pediatrics – Istanbul – Turkey.; 5 Demiroğlu Bilim University – Department of Physiology – Istanbul – Turkey.

**Keywords:** Tamoxifen, Caffeine, Fatty Liver

## Abstract

**Purpose:**

Tamoxifen, a widely used drug for breast cancer treatment, is associated with adverse effects on the liver, including the development of fatty liver. This study aimed to investigate the potential protective effect of caffeine against tamoxifen-induced fatty liver in Wistar rats.

**Methods:**

Rats were divided into normal control, tamoxifen + saline, and tamoxifen + caffeine. Plasma samples were assessed for biochemical markers related to oxidative stress, inflammation, liver function, and cell damage. Additionally, liver histopathology was examined to quantify the extent of fatty infiltration.

**Results:**

In the tamoxifen + saline group, elevated levels of plasma malondialdehyde (MDA), tumor necrosis factor-alpha (TNF-α), alanine aminotransferase (ALT), cytokeratin 18, and soluble ST2 were observed compared to the normal control group, indicating increased oxidative stress, inflammation, and liver injury (*p* < 0.01). Moreover, histopathological examination revealed a significant increase in fatty infiltration (*p* < 0.001). However, in the tamoxifen + caffeine group, these markers were markedly reduced (*p* < 0.05, p < 0.01), and fatty infiltration was significantly mitigated (*p* < 0.001).

**Conclusions:**

The findings suggest that caffeine administration attenuates tamoxifen-induced fatty liver in rats by ameliorating oxidative stress, inflammation, liver injury, and cell damage. Histopathological evidence further supports the protective role of caffeine. This study highlights the potential of caffeine as a therapeutic intervention to counter tamoxifen-induced hepatic complications, contributing to the optimization of breast cancer treatment strategies.

## Introduction

Among women, breast cancer is one the most prevalent form of cancer and ranks as the second leading cause of cancer-related fatalities. Around 70% of breast cancer patients have hormone receptor-positive tumors^1^. Hormonal therapies are advised for those with hormone receptor-positive breast cancer to reduce the risk of disease recurrence and enhance overall survival. Tamoxifen and aromatase inhibitors are the primary drugs utilized in hormonal therapy for breast cancer patients^2^.

Tamoxifen is a nonsteroidal estrogen receptor antagonist, is relatively cheaper, and has fewer side effects than aromatase inhibitors. Therefore, it is the first choice of adjuvant hormone therapy in premenopausal patients^3^.

The most common side effect of tamoxifen use is fatty liver^4^. Approximately 35% of breast cancer patients who have taken oral tamoxifen for 3–5 years may develop tamoxifen-induced fatty liver, which can be detected on yearly computed tomography scans^4^^-^ 6. While it is known that tamoxifen use can lead to liver cirrhosis if fatty liver disease is not controlled, it is one of the most severe rate-limiting steps in treatment maintenance^7^.

Fatty liver disease is the most common disease, which occurs in one out of every five people in the normal population^7^. Although its etiology is not known precisely, it is a condition that results in ballooning in hepatocytes, lobular inflammation, and ultimately fibrosis after fat oxidation disorder^8^. Oxidative stress and many inflammatory factors, including interleukin 1 (IL-1), tumor necrosis factor-alpha (TNF-α), interleukin 6 (IL-6), suppression of tumorigenicity (ST2), nuclear factor kappa-B (NF-κB) are thought to be involved in the pathogenesis^9^^-^ 12.

There are many different opinions about the mechanism of tamoxifen-associated fatty liver disease. The most accepted of these views is the increase in lipogenesis. With this increased lipogenesis, oxidative stress occurs, which releases inflammatory cytokines. These inflammatory cytokines disrupt lipid oxidation in the liver and cause fat deposition in hepatocytes^5^^,^ 13 ^-^ 19.

TNF-α, cytokeratin 18, soluble suppression of tumorigenicity (sST2), and malondialdehyde (MDA) molecules, which we examined in our study, are cytokines with increased levels in fatty liver disease. TNF-α is a proinflammatory cytokine produced primarily by monocytes and macrophages and is the main cytokine with increased levels in acute liver disease and fatty liver disease^19^^-^ 22. Cytokeratin 18 is an essential intermediate filament protein in hepatocytes and increases in steatosis^23^. This increase is correlated with the level of adiposity in the liver, and the level of cytokeratin 18 increases as the level of steatosis increases^23^. MDA is one of the end products of the peroxidation of polyunsaturated fatty acids in cells. An increase in free radicals leads to excessive production of MDA. MDA level is a marker of oxidative stress and antioxidant status in patients with cancer^24^. Oxidative stress levels can be evaluated by measuring MDA levels in biological fluids such as blood, urine, or saliva. The other molecule we examined in our study was suppression of ST2. ST2 is a cytokine with two isoforms, sST2 and a transmembrane receptor (ST2 ligand, or ST2L). Its level increases in acute and chronic liver damage^25^.

Caffeine, which we investigated its protective effect on tamoxifen-associated adiposity of the liver, is a potent antagonist of adenosine receptors in the central and peripheral nervous system and inhibits the release of excitatory neurotransmitters^26^. Caffeine has been shown to increase glutathione and S-transferase, which are protective enzymes against oxytocic stress, in *in-vitro* studies^27^. It is estimated that caffeine has a protective effect on the adiposity of the liver by decreasing oxidative stress^28^^,^ 29.

This study examined the oxidative stress associated with tamoxifen and the cytokines that increase with this stress. In addition, we tried to prove that caffeine decreases fat deposition in the liver by giving caffeine to a group that was given tamoxifen.

## Methods

First, the Animal Ethics Committee of Demiroğlu Science University, Istanbul, Turkey, gave ethical approval (Science University, Ethical number: 1823082901/18.01.2023). The rats utilized in the experiment were acquired from the Experimental Animal Laboratory of Science University Gebze, Istanbul, Turkey.

Twenty-one adult female Wistar rats, with an average weight of 200–210 g, were utilized for the experiment. The animals were confined in enclosures and subjected to controlled environmental conditions, including a 12-hour alternation between light and darkness while being kept at a consistent room temperature of 22 ± 2 °C. Throughout the study, the subjects were provided with a standard pellet diet and had unrestricted access to tap water. All chemical substances were acquired from Sigma-Aldrich Inc., unless explicitly stated otherwise.

### Experimental procedure

The experimental procedure involved 21 rats in the study. Seven rats were selected to serve as the normal control group. No medication was administered to this cohort.

Fourteen rats were administered tamoxifen orally via gavage at 8 mg per kilogram daily for six weeks. This was done to induce a model of tamoxifen-induced fatty liver. The rats that were administered tamoxifen were separated into two distinct groups. Seven rats were assigned to group 1, which received a daily intraperitoneal (i.p.) administration of 1 mL/kg/day % 0.9 NaCl saline for six weeks. Similarly, group 2, consisting of seven rats, received a daily i.p. administration of 25 mg/kg/day caffeine for six weeks.

Upon completing the study, the rats were euthanized using a high dose of anesthesia by applying the cervical dislocation procedure. Blood samples were obtained via cardiac puncture for biochemical analysis, while organs underwent histopathological examination.

### Measurement of plasma lipid peroxidation

Plasma lipid peroxidation, specifically MDA levels, was quantified in plasma samples by assessing MDA concentrations as thiobarbituric acid reactive substances (TBARS). Concisely, the experimental procedure involved the addition of trichloroacetic acid and TBARS reagent to the plasma samples, followed by thorough mixing and subsequent incubation at the temperature of 100 °C for 60 min. After cooling on ice, the samples underwent centrifugation at the speed of 3,000 revolutions per min for 20 min. Subsequently, the absorbance of the resulting supernatant was measured at a wavelength of 535 nanometers. The MDA levels were quantified in nanomolar (nM) units, with tetraethoxypropane as the calibration standard.

### Measurement of plasma TNF-α, alanine aminotransferase, cytokeratin 18, soluble ST2

TNF-α plasma levels, alanine aminotransferase (ALT), cytokeratin 18, and soluble ST2 were quantified by commercially available enzyme-linked immunosorbent assay (ELISA) kits.

### Histopathological examination of liver

The histopathological examination of the liver involves the microscopic analysis of tissue samples obtained from the liver to assess and diagnose various pathological conditions affecting this organ. In order to conduct histological and immunohistochemical investigations, the animals were administered anesthesia through i.p. injection of ketamine (100 mg/kg) and xylazine (10 mg/kg). Subsequently, they were perfused with a solution of 4% formaldehyde in 0.1 M phosphate-buffered saline (PBS), with a total volume of 200 mL. Liver sections of 4-μm thickness fixed with formalin were subjected to staining using hematoxylin and eosin. The sections were captured using an Olympus C-5050 digital camera affixed to an Olympus BX51 microscope.

The morphological evaluation was conducted using a computerized image analysis system (Image-Pro Express 1.4.5, Media Cybernetics, Inc., United States of America). Ten microscopic fields per section were examined at a magnification of ×20. The observer conducting the evaluation was unaware of the study group. The fatty infiltration percentage of liver sections from all rats in each experimental group was assessed.

### Statistical analysis

The statistical analysis was conducted using Statistical Package for the Social Sciences version 15.0 for Windows. The parametric variables were compared using statistical tests such as the Student’s t-test and analysis of variance. In addition, the comparison between groups of nonparametric variables was conducted using the Mann–Whitney’s U test. Furthermore, the Shapiro–Wilk’s test was employed to distinguish between parametric and nonparametric data. The findings are reported as mean plus standard error of the mean (SEM). A significance level of *p* < 0.05 was deemed acceptable for establishing statistical significance.

## Results

Table 1 shows the levels of plasma markers associated with oxidative stress, inflammation, liver function, and cell damage. In the tamoxifen + saline group, there was a significant increase in plasma MDA level (57.2 ± 3.3 nM) compared to the normal control group (36.1 ± 2.1 nM), indicating elevated oxidative stress (*p* < 0.01). Similarly, plasma TNF-α level was significantly elevated in the tamoxifen + saline group (35.1 ± 1.06 pg/mL) compared to the normal control group (16.5 ± 0.3 pg/mL), indicating increased inflammation (*p* < 0.01). Plasma ALT level, a marker of liver injury, was also significantly higher in the tamoxifen + saline group (63.2 ± 2.6 IU/L) compared to the normal control group (41.5 ± 1.8 IU/L) (*p* < 0.01). The plasma levels of cytokeratin 18 and sST2, markers of cell damage, were elevated in the tamoxifen + saline group (*p* < 0.01).

**Table 1:** The levels of plasma markers associated with oxidative stress, inflammation, liver function, and cell damage@.

	Normal control	Tamoxifen + saline	Tamoxifen + caffeine
Plasma MDA (nM) level	36.1 ± 2.1	57.2 ± 3.3 *	39.5 ± 0.6 #
Plasma TNF-α (pg/mL) level	16.5 ± 0.3	35.1 ± 1.06 *	23.8 ± 2.5 #
Plasma ALT (IU/L) level	41.5 ± 1.8	63.2 ± 2.6 *	44.9 ± 3.7 #
Plasma cytokeratin 18 (pg/mL) level	0.77 ± 0.2	1.65 ± 0.1 *	0.98 ± 0.1 #
Plasma soluble ST2 (pg/mL) level	0.94 ± 0.09	2.24 ± 0.1 *	1.15 ± 0.1 #
Liver histopathological examination for fatty infiltration (%)	1.2 ± 0.1	69.5 ± 5.6 **	8.3 ± 1.5 ##

MDA: malondialdehyde; TNF-α: tumor necrosis factor-alpha; ALT: aminotransferase;

@ results were presented as mean ± standard error of the mean. Statistical analyses were performed by one-way analysis of variance test;

* *p* < 0.01

** *p* < 0.001 (different from control group)

# *p* < 0.05

# *p* < 0.001 (different from tamoxifen and saline group).

Source: Elaborated by the authors.

However, in the tamoxifen + caffeine group, the levels of these markers were notably lower than those in the tamoxifen + saline group. Plasma MDA, TNF-α, ALT, cytokeratin 18, and soluble ST2 levels in the tamoxifen + caffeine group were 39.5 ± 0.6 nM, 23.8 ± 2.5 pg/mL, 44.9 ± 3.7 IU/L, 0.98 ± 0.1 pg/mL, and 1.15 ± 0.1 pg/mL, respectively. These values were significantly different from those in the tamoxifen + saline group, indicating that caffeine administration mitigated oxidative stress, inflammation, and cell damage induced by tamoxifen (*p* < 0.05, *p* < 0.01) (Table 1).

**Figure 1:**
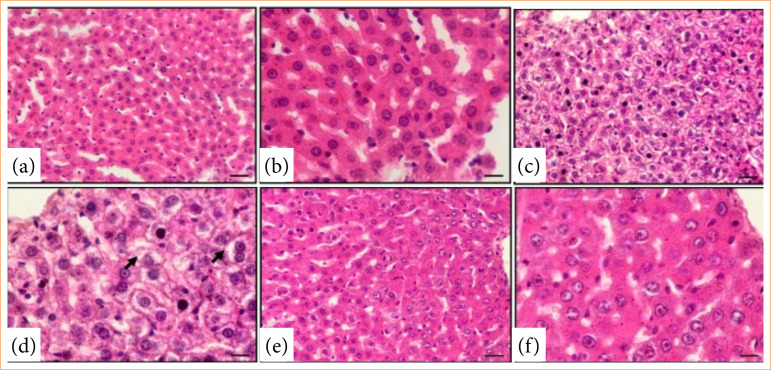
Liver histolopathology hematoxylin and eosin stain (x20 and x40 magnification). (a and b) Normal group rats have normal liver; (c and d) tamoxifen and saline group rats have lipid droplets in hepatocytes (arrow); (e and f) tamoxifen + caffeine group rats have decreased have lipid droplets in hepatocytes.

Source: Elaborated by the authors.

Furthermore, liver histopathological examination revealed a substantial increase in fatty infiltration in the tamoxifen + saline group (69.5 ± 5.6%) compared to the normal control group (1.2 ± 0.1%) (*p* < 0.001). In contrast, the tamoxifen + caffeine group showed a significant reduction in fatty infiltration (8.3 ± 1.5%) compared to the tamoxifen + saline group (*p* < 0.001) (Table 1, Fig. 1).

Figure 1 displays representative liver histopathology images supporting the biochemical findings. Normal control rats exhibited normal liver histology, while rats in the tamoxifen + saline group showed lipid droplets in hepatocytes, indicating fatty liver development. Rats in the tamoxifen + caffeine group demonstrated decreased lipid droplets in hepatocytes, suggesting the protective effect of caffeine against tamoxifen-induced fatty liver (Fig. 1).

## Discussion

Tamoxifen is an effective drug for treating hormone receptor-positive breast cancer^30^. Although generally well tolerated, fatty liver disease commonly occurs in patients receiving tamoxifen^31^^,^ 32. In a meta-analysis examining fatty liver in breast cancer patients with and without tamoxifen use, statistically significantly more fatty liver in patients taking tamoxifen was observed^14^. The rate of tamoxifen-associated adiposity of the liver was higher in obese individuals^33^. In a study by Yan et al.^16^, it was found that tamoxifen-associated adiposity of the liver was more frequent in patients with a body mass index higher than 22 kg/m^2^.

The mechanism of how tamoxifen causes fatty liver disease is not yet fully understood. The most widely accepted hypothesis is that oxidative stress caused by increased lipogenesis is the triggering factor. Oxytadic stress releases inflammatory cytokines. These inflammatory cytokines disrupt lipid oxidation in the liver and lead to fat deposition in hepatocytes^5^^,^ 12 ^-^ 16.

In a study by Lv et al.^34^, they describe how caffeine has the potential to shield against liver damage caused by alcohol by decreasing oxidative stress and inflammation, providing a new and unique method of protection.

TNF-α is the first cytokine increased in Kuppfer cell adiposity^35^. Many pieces of evidence have shown a positive correlation between TNF-α and adiposity in the liver^36^. In addition, studies have also shown that TNF-α can be used as a predictive factor for the development of adiposity^37^. Moreover, Horrigan et al.^38^ found that human blood exposed to caffeine at concentrations commonly found in human consumption consistently inhibits the production of TNF-α, a cytokine, through the action of cyclic AMP/protein kinase, a route or course that allows for passage or progress. In this study, the TNF-α level was 35.1 ± 1.06 in the tamoxifen + saline group and was statistically significantly higher than the control group. This statistical significance was consistent with the literature.

MDA is one of the end products of the peroxidation of polyunsaturated fatty acids in cells and known as a marker of oxidative stress and antioxidant status^24^. The relation between caffeine and MDA is described in a study by Amat et al.^39^. They claim that consuming robust coffee can elevate MDA levels in individuals in good health, potentially suggesting the presence of oxidative stress in the body. On the other hand, the antioxidant component called caffeic acid phenethyl ester CAPE, found in propolis extract, can potentially safeguard the spinal cord from ischemia-reperfusion injury in rabbits without causing any additional harm to the tissue^40^. In this study, the MDA molecule we used as a lipid peroxidation product was significantly higher in the tamoxifen + saline group than in the control group. This elevation is similar to a previous study^41^.

The soluble ST2 molecule is a cytokine belonging to the IL-1 group, which has been used in recent years. Its level increases in fatty liver disease, toxic hepatitis, autoimmune hepatitis, and fibrosis of the liver^41^^-^ 43. When we analyzed the level of this new molecule soluble ST2 in tamoxifen-associated adiposity of the liver, the level of soluble ST2 was 0.94 ± 0.09 pg/mL in the control group and 2.24 ± 0.1 pg/mL in the tamoxifen + saline group, which was statistically significantly higher. We found that soluble ST2, which we measured at a significantly high value, is a marker that can be used in tamoxifen-associated fatty liver tumors, just as in other types of liver damage.

Another molecule we measured in our study was cytokeratin 18. Cytokeratin 18 is an intermediate filament in hepatocytes and is increased in hepatosteatosis. Data from two meta-analyses in which this molecule was analyzed showed that it could be used in fatty liver disease with a mean sensitivity and specificity of 66–78% and 82–87%, respectively^20^^,^ 44. Moreover, measuring the CK-18 concentration can be used as a biomarker to evaluate the effectiveness of treatment and enhance the management of nonalcoholic fatty liver disease in individuals with type-2 diabetes mellitus^45^. This study showed a significant elevation of cytokeratin 18 in the tamoxifen + saline group compared to the control group.

ALT is the most commonly used biochemical marker for diagnosing and following up liver lipidaemia during tamoxifen treatment^46^^,^ 47. An increase of ALT ≥ 1.5 times the upper limit of normal in tamoxifen-treated patients is suggestive of lipidaemia of the liver. Our study showed an average 1,5 times increase in the tamoxifen + saline group compared to the control group. It is one of the most straightforward, but most significant signs of the healing effect of caffeine.

There are very few therapeutic options to reduce fatty liver disease. Clinical studies and meta-analyses show that caffeine protects fatty liver, fibrosis, cirrhosis, and hepatocellular carcinoma^48^^-^ 51. Experimental studies have shown that caffeine inhibits hepatic stellate cell activity by blocking A2A receptors. The same study showed caffeine might also positively affect angiogenesis and liver hemodynamics^52^. The favorable effects of caffeine on hepatic steatosis have been demonstrated in experimental and observational studies^52^. Considering these studies, we tried to show the positive effect of caffeine on tamoxifen-associated lipidosis of the liver with biochemical and histopathological evidence.

The rising prevalence of hepatosteatosis and its strong correlation with metabolic disorders such as obesity, diabetes, and hyperlipidemia have made it a significant area of research^53^. In this study, a histopathological examination of the liver showed marked lipidosis in the tamoxifen + saline group. In the tamoxifen + saline group, 69.5 ± 5.6% of KC steatosis was observed, whereas it was 8.3 ± 1.5% in the tamoxifen + caffeine group. Caffeine significantly decreased tamoxifen-induced fatty liver tumors. Compared to the tamoxifen + saline group, plasma MDA, TNF-alpha, ALT, cytokeratin 18, and soluble ST2 levels were significantly lower in the tamoxifen + caffeine group. In conclusion, we found that caffeine alleviated histopathological and biochemical tamoxifen-induced lipidosis of the liver.

## Conclusion

No study to our knowledge shows that caffeine attenuates tamoxifen-induced fatty liver inflammation. In this respect, this study, in which we have shown that caffeine attenuates tamoxifen-induced fatty liver inflammation, is valuable.

## Data Availability

The datasets generated and analyzed during the current study are available from the corresponding author upon reasonable request.
